# Time-efficient physical activity interventions to reduce blood pressure in older adults: a randomised controlled trial

**DOI:** 10.1093/ageing/afaa211

**Published:** 2020-10-17

**Authors:** Philip J J Herrod, Jonathan N Lund, Bethan E Phillips

**Affiliations:** Medical Research Council-Versus Arthritis Centre for Musculoskeletal Ageing Research, and NIHR Nottingham Biomedical Research Centre, University of Nottingham, Royal Derby Hospital Centre, Derby, UK; Department of Anaesthetics and Surgery, Royal Derby Hospital, Derby, UK; Medical Research Council-Versus Arthritis Centre for Musculoskeletal Ageing Research, and NIHR Nottingham Biomedical Research Centre, University of Nottingham, Royal Derby Hospital Centre, Derby, UK; Department of Anaesthetics and Surgery, Royal Derby Hospital, Derby, UK; Medical Research Council-Versus Arthritis Centre for Musculoskeletal Ageing Research, and NIHR Nottingham Biomedical Research Centre, University of Nottingham, Royal Derby Hospital Centre, Derby, UK

**Keywords:** exercise, blood pressure, ageing, health, static, older people

## Abstract

**Background:**

Hypertension is a risk factor for both cardiovascular and cerebrovascular disease, with an increasing incidence with advancing patient age. Exercise interventions have the potential to reduce blood pressure in older adults, however, rates of exercise uptake and adherence are low, with ‘lack of time’ a commonly cited reason. As such, there remains the need for time-efficient physical activity interventions to reduce blood pressure in older adults.

**Objective:**

To compare the effect of three, novel time-efficient physical activity interventions on resting blood pressure in older adults.

**Methods:**

Forty-eight, healthy, community-dwelling older adults (mean age: 71 years) were recruited to a 6-week randomised control trial. Resting blood pressure was measured before and after one of three supervised, time-efficient interventions: high-intensity interval training (HIIT) on a cycle ergometer; isometric handgrip training (IHG); unilateral, upper limb remote ischaemic preconditioning (RIPC) or non-intervention control.

**Results:**

Both HIIT and IHG led to a statistically significant reduction in resting systolic blood pressure (SBP) of 9 mmHg, with no significant change in the RIPC or control groups. There was no change in diastolic blood pressure or pulse pressure in any group.

**Conclusions:**

Supervised HIIT or IHG using the protocols described in this study can lead to statistically significant and clinically relevant reductions in resting SBP in healthy older adults in just 6 weeks.

## Key points

- Six-week high-intensity interval training can reduce the systolic blood pressure (SBP) of older adults.
- Six-week low-exertion isometric handgrip training can also reduce the SBP of older adults.
- The reductions in SBP elicited by both these interventions are of a clinically relevant magnitude.

## Introduction

The control of hypertension is key to the management of cardiovascular risk throughout national guidelines [1]. Meta-analysis of antihypertensive therapy demonstrates that a 10 mmHg reduction in systolic blood pressure (SBP) is associated with a vast reduction in cardiovascular disease risk [2]. Although lifestyle (namely diet and exercise based) recommendations are made in guidelines, their applicability to older adults remains untested, with the majority of evidence from young adults [1]. This is despite the well-described association between advancing age and hypertension, with prevalence increasing from 8% of men and 2% of women in the 16–24 years age range to 66% of men and 78% of women aged 75 years and over [3]. In addition, uptake and adherence of exercise is strikingly poor across the population with only 10–15% of older adults achieving government recommendations for physical activity [4], with ‘lack of time’ a commonly cited barrier to achieving this [5,6]. As such, there remains the need for an effective, time-efficient lifestyle intervention to reduce blood pressure in older adults. A previous systematic review of non-pharmacological interventions in older adults demonstrated the blood pressure lowering effects of aerobic exercise, dynamic resistance exercise and combined training when carried out by older adults [7]. However, the majority of interventions lasted 3 months or more, with sessions often exceeding 60 min in duration. Previous studies have identified that high-intensity interval training (HIIT) [8], isometric handgrip training (IHG) [9] and remote ischaemic preconditioning (RIPC) [10] may also have utility in this regard with the potential added benefit of being time-efficient; however, all have yet to be assessed in a randomised controlled trial (RCT) of older adults over a short time frame.

## Methods

Ethical approval was obtained to recruit healthy (American Society of Anesthesiologists grade 1 or 2) adults aged 65–85 years to participate in a trial of 6-week fully supervised HIIT, RIPC, IHG or non-intervention control (CON) (NCT03019146). All participants gave written informed consent. Participants already prescribed antihypertensives were included, providing no alterations were made between 3 months prior to enrolment and study close. The primary outcome of the study was resting SBP.

Resting blood pressure (BP) was measured at the start and end of the study using oscillometry (Datascope Trio, New Jersey, USA) and a blood pressure cuff of appropriate size, according to current clinical guidelines [11] using the subject’s dominant arm. Three measurements of blood pressure were made on each study day, in a seated position, at a standardised time, after a period of 30-minute observed rest in a temperature-controlled room. The arithmetic mean of the three measurements was taken as the BP [12].

After their baseline measurement, participants were randomised to either HIIT, RIPC, IHG or control using computerised randomisation (sealedenvelope.com), stratified to baseline SBP (<140 mmHg or ≥ 140 mmHg) and antihypertensive medication.

### Interventions

Subjects assigned to an intervention group attended for supervised training three times each week for 6 weeks, with each session lasting approximately 15 min.

HIIT subjects first performed a ramped incremental exercise test to volitional exhaustion in order to determine their training intensity. Each training session comprised a 2-minute warm-up, followed by five 1-minute intervals at 90–110% of peak power output interspersed with 90 s of unloaded cycling and a final 3.5-minute recovery phase. After 3 weeks of HIIT, intensity was increased by 10% for the final 3 weeks.

RIPC subjects were conditioned with a blood pressure cuff (Welch Allyn, NY, USA), placed on their dominant arm and inflated to 200 mmHg for 3 min before deflating. This was repeated three times in each session with 3-minute rest between each inflation.

IHG subjects completed four 2-minute repetitions of isometric handgrip at 30% of maximum voluntary contraction (MVC) on an electronic hand dynamometer (Camry EH101, Zhongshan Camry Electronic Co. Ltd, Guangdong, China) using their dominant hand, with 2-minute rest between each contraction. MVC was measured according to the American Society of Hand Therapists clinical assessment recommendations, with the participant seated, the shoulder adducted and neutrally rotated and the elbow flexed to 90 degrees [13]. Each participant was given three attempts to measure an MVC using the same dynamometer as used for training, with 1-minute rest between each attempt. The MVC was recorded as the highest reading obtained.

### Statistical analysis

An *a priori* power calculation determined a sample size of 48 was required for 90% power to detect a reduction in SBP of 10 mmHg. All calculations were performed using Graphpad Prism Version 7.02 (California, USA). Data are presented as mean [standard deviation SD]. Outcome data were compared using two-way analysis of variance with Sidak’s multiple comparisons post-hoc testing. Significance was taken as an alpha of *P* < 0.05.

## Results

**Figure 1:**
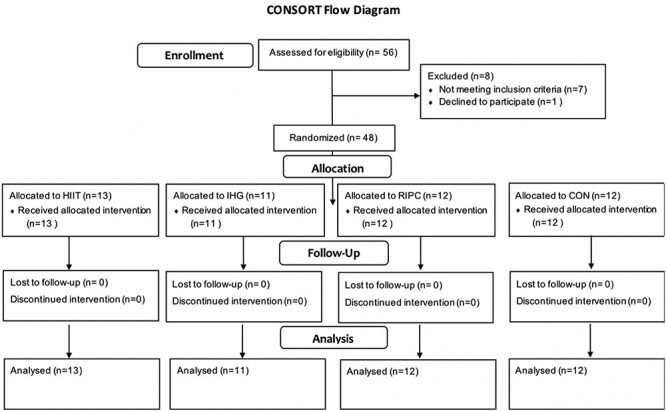
CONSORT diagram.

Forty-eight participants of mean age 71(4) years (46% male) were randomised and none were lost to follow-up. Users of anti-hypertensive medication were equally distributed throughout the groups (3 HIIT, 3 IHG, 4 RIPC and 5 CON), with equal sex distribution (5 males HIIT, 4 IHG, 6 RIPC and 7 CON). Mean (SD) training compliance was 99(3)%, and there were no adverse events. The study consort diagram is displayed in Figure 1.

**Figure 2:**
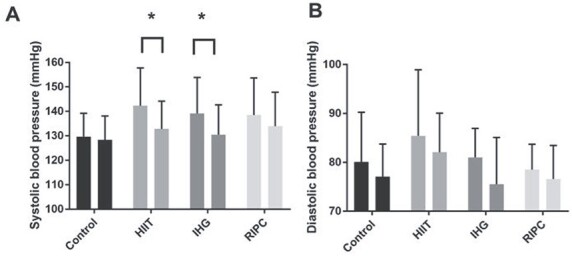
Systolic (A) and diastolic (B) blood pressure before (left bar) and after (right bar) a 6-week intervention period of no-intervention control, HIIT, IHG or RIPC. Values are mean (SD). * = *P* < 0.05 vs. pre-intervention values.

Data for both resting SBP and diastolic blood pressure (DBP) at baseline and after intervention are displayed in Figure 2. There was no significant difference between the groups at baseline for either SBP (*P* = 0.15) or DBP (*P* = 0.30). For SBP, there was a main effect of time (*P* < 0.001) and a significant group x time interaction (*P* = 0.04), with significant reductions in both the HIIT (142(15) vs. 133(11); −9(9) mmHg, *P* < 0.001) and IHG (139(15) vs. 130(12); −9(9)mmHg, *P* = 0.002) groups. There was no significant change in either the RIPC (138(15) vs. 134(14); −4(5), *P* = 0.17) or control (130(10) vs. 128(10); −1(6), *P* = 0.96) groups. For DBP, there was a main effect of time (*P* = 0.01), however, no group x time interaction (*P* = 0.83). There was no effect of either time (*P* = 0.09) or group x time interaction on pulse pressure (*P* = 0.33).

## Discussion

This study has demonstrated that 6 weeks of fully supervised HIIT or IHG can reduce the resting SBP of older adults by an average of 9 mmHg. This reduction is of a clinically relevant magnitude, likely to substantially reduce a patient’s risk of myocardial infarction by as much as 21% and of stroke by 41% [2]. The fact that these interventions are time efficient, with each session lasting only 15 min, and effective in as short a time frame as 6 weeks, could lead to their use as either a general lifestyle intervention that could be prescribed by general practitioners to reduce general cardiovascular risk status, or as specific treatment of hypertension [14]. Previous studies using HIIT in both young healthy subjects [15] and middle-aged hypertensive subjects [16] have shown that HIIT can reduce blood pressure, and it appears from our findings that this is transferrable to older age. Of note, the only other study reporting BP results from a short-term HIIT protocol in older adults (albeit in men only), showed a similar magnitude of change [17]. This cohort study by Grace *et al*., asked their volunteers, with a mean age of 62 years, to perform nine sessions of HIIT over a 6-week intervention period, with each session comprising six 30-second sprints on a cycle ergometer, at 40% of peak power output determined using a 6-second test (with a reported training mean training intensity at 141% of the W peak at volitional exhaustion during an incremental cycle test [18]), interspersed with 3-minute resting intervals. Thus, it would appear that our reduced-intensity 5 × 1-minute HIIT protocol can achieve similar reductions in SBP, with a shorter session duration and in a mixed sex, older cohort.

The effect of the IHG protocol on SBP in this study was approximately equal to the mean change found in the meta-analysis of the two previous trials of IHG in older adults, which were conducted over 8 [19] and 10 weeks [20], respectively. It may therefore be that maximal improvements in SBP are achieved by 6 weeks, with prolonged training unable to produce further benefit.

It would appear that RIPC conducted using a unilateral arm 3 × 3-minute occlusion protocol does not have any effect on BP. This was the first RCT to examine the effects of unilateral upper limb RIPC with only 3-minute ischaemic periods in older adults and did not reproduce the significant reduction in resting BP found by Jones *et al*. [21]. It may be that reducing the ischaemic time periods to 3 min (from 5 min) is responsible for this discrepancy, or that the effect of RIPC on the younger subjects in Jones’ study is not transferrable to older age.

As with almost all research, we must consider limitations to our study design. Despite stratified randomisation for hypertension and medication status, our relatively small sample size led to some baseline imbalances in starting blood pressure and precluded across-group effect comparisons. In addition, no data on compliance with antihypertensive medication was recorded, with participants simply instructed not to make any changes to this. Other key limitations of this study are that only two participants were aged over 80 and that none had significant comorbidity, limiting the interpretation of this study with regards to these groups.

It is, however, promising that two time-efficient interventions, one of which is relatively ‘static’ in nature, appear to have potential as tools to reduce blood pressure in older adults. Future work should examine the possibility of using HIIT and IHG interventions as home-based, unsupervised interventions and conduct longer duration follow-up to assess the durability of these adaptions.
